# Aerobic Exercise Training Selectively Changes Oxysterol Levels and Metabolism Reducing Cholesterol Accumulation in the Aorta of Dyslipidemic Mice

**DOI:** 10.3389/fphys.2017.00644

**Published:** 2017-09-05

**Authors:** Guilherme Silva Ferreira, Paula R. Pinto, Rodrigo T. Iborra, Vanessa Del Bianco, Monique Fátima Mello Santana, Edna Regina Nakandakare, Valéria S. Nunes, Carlos E. Negrão, Sergio Catanozi, Marisa Passarelli

**Affiliations:** ^1^Laboratorio de Lipides, Laboratorio de Investigaçao Medica – 10 (LIM-10), Hospital das Clinicas (HCFMUSP), Faculdade de Medicina, Universidade de Sao Paulo Sao Paulo, Brazil; ^2^Unidade de Reabilitação Cardiovascular e Fisiologia do Exercício, Instituto do Coração InCor da Faculdade de Medicina, Universidade de São Paulo São Paulo, Brazil

**Keywords:** aerobic exercise training, atherosclerosis, dyslipidemia, oxysterol, reverse cholesterol transport

## Abstract

**Background:** Oxysterols are bioactive lipids that control cellular cholesterol synthesis, uptake, and exportation besides mediating inflammation and cytotoxicity that modulate the development of atherosclerosis. Aerobic exercise training (AET) prevents and regresses atherosclerosis by the improvement of lipid metabolism, reverse cholesterol transport (RCT) and antioxidant defenses in the arterial wall. We investigated in dyslipidemic mice the role of a 6-week AET program in the content of plasma and aortic arch cholesterol and oxysterols, the expression of genes related to cholesterol flux and the effect of the exercise-mimetic AICAR, an AMPK activator, in macrophage oxysterols concentration.

**Methods:** Sixteen-week old male apo E KO mice fed a chow diet were included in the protocol. Animals were trained in a treadmill running, 15 m/min, 5 days/week, for 60 min (T; *n* = 29). A control group was kept sedentary (S; *n* = 32). Plasma lipids and glucose were determined by enzymatic techniques and glucometer, respectively. Cholesterol and oxysterols in aortic arch and macrophages were measured by gas chromatography/mass spectrometry. The expression of genes involved in lipid metabolism was determined by RT-qPCR. The effect of AMPK in oxysterols metabolism was determined in J774 macrophages treated with 0.25 mM AICAR.

**Results:** Body weight and plasma TC, TG, HDL-c, glucose, and oxysterols were similar between groups. As compared to S group, AET enhanced 7β-hydroxycholesterol (70%) and reduced cholesterol (32%) in aorta. In addition, exercise increased *Cyp27a1* (54%), *Cd36* (75%), *Cat* (70%), *Prkaa1* (40%), and *Prkaa2* (51%) mRNA. In macrophages, the activation of AMPK followed by incubation with HDL_2_ increased *Abca1* (52%) and *Cd36* (220%) and decrease *Prkaa1* (19%), *Cyp27a1* (47%) and 7α-hydroxycholesterol level.

**Conclusion:** AET increases 7β-hydroxycholesterol in the aortic arch of dyslipidemic mice, which is related to the enhanced expression of *Cd36*. In addition, the increase and reduction of *Cyp27a1* and *Cyp7b1* in trained mice may contribute to enhance levels of 27-OH C. Both oxysterols may act as an alternative pathway for the RCT contributing to the reduction of cholesterol in the aortic arch preventing atherogenesis.

## Introduction

Oxysterols are a broad group of bioactive lipids derived from enzymatic and non-enzymatic oxidation of the cholesterol molecule that takes place both intra and extracellularly (Brown and Jessup, 2009). Side-chain oxysterols such as, 24S-hydroxycholesterol, 25-hydroxycholesterol (25-OH C), and 27- hydroxycholesterol (27-OH C), are enzymatically produced and efficient mediate cholesterol synthesis and uptake by regulating the activation of SREBP and the expression of the HMG-CoA reductase and low-density lipoprotein (LDL) receptor, respectively (Radhakrishnan et al., 2008). These oxysterols also maintained cell cholesterol homeostasis by the induction of *ABCA1* and *ABCG1* gene transactivation, favoring the exportation of cholesterol excess to apo A-I and mature high-density lipoprotein (HDL) and the cholesterol flow through the reverse cholesterol transport (RCT; Venkateswaran et al., 2000). The RCT is an anti-atherogenic system that drives cholesterol from the arterial wall to the liver allowing its excretion into bile and feces.

7-ketocholesterol (7-KC) and 7β-hydroxycholesterol (7β-OH C) may be directly involved in the atherogenesis considering their role in inflammation, endoplasmic reticulum stress and apoptosis and its prevalence in atherogenic oxidized LDL (Gargiulo et al., 2016). Conversely, they can be exported from cells via ABCG-1 transporter (Terasaka et al., 2007). In apo E knockout mice (apo E KO) 80% of the total amount of arterial oxysterols are represented by the compounds derived from carbon-7 cholesterol oxidation (7-KC > 7β-OH C > 7α-OH C; Vaya et al., 2001). In the same animal model, Shentu et al. (2012) demonstrated that 7α-OH C and 7β-OH C were the most abundant forms in the arterial compartment.

Because of their higher polarity as compared to cholesterol the oxysterols diffuse much better through phospholipid membranes. The half-life for 7α-OH C and 7β-OH C exchange between lipid vesicles is 100 times higher than that observed for cholesterol (Kan et al., 1992). Babiker et al. (1997) and Lund et al. (1996) observed that macrophages eliminate large amounts of cholesterol in 27-OH C form, which is mediated by HDL, acting as an additional pathway for the RCT.

Physical exercise is a well-established non-pharmacological strategy in the management of lipid metabolism, reducing plasma triglyceride (TG) concentration and small dense LDL and increasing HDL cholesterol and RCT (Kodama et al., 2007; Rocco et al., 2011; Ghafouri et al., 2015; Pinto et al., 2015). Together with its beneficial effects in other risk factors for cardiovascular disease, exercise training has an important role in preventing, reducing and stabilizing atherosclerotic lesion.

The adenosine monophosphate-activated protein kinase (AMPK) is a classical target of exercise in many tissues including the arterial wall, acting as a sensor of energy levels (Kim et al., 2014). Besides inducing mitochondrial biogenesis, fatty acid, and glucose oxidation, recent studies suggest that AMPK plays an important role in atherogenesis (Li et al., 2010a,b; Chen et al., 2016). There are no data in literature on the role played by exercise training in the amount of cholesterol and oxysterols in plasma and arterial wall and how this relates *in vivo* with the expression of genes involved in oxysterols metabolism in dyslipidemic apo E KO mice and *in vitro* with AMPK activation in macrophages. Arterial macrophages are intimately related to the development of atherosclerotic lesion by mediating the uptake of modified LDL but also excess lipid efflux by the RCT. We then analyzed the role of a 6-week AET in the distribution of oxysterol subspecies and total cholesterol (TC) in plasma and aorta of apo E KO mice and the effect of AMPK activation in J774 macrophages in cellular content and efflux of oxysterols.

## Materials and methods

### Animals

Apo E KO mice were purchased from Jackson Laboratory (Bar Harbor, Maine, USA). Animals were kept in a conventional housing at 22 ± 2°C in a 12 h light/12 h dark cycle with free access to chow diet (Nuvilab-Nuvital, São Paulo, Brazil) and water. Protocols with animals were approved by the Institutional Animal Care and Research Advisory Committee (Hospital das Clinicas HCFMUSP, Faculdade de Medicina, Universidade de São Paulo, São Paulo,—n°074/14). Food consumption was evaluated weekly throughout the study and calculated as (weight food given—weight food uneaten)/number of animal per cage/day.

### Training protocol

Sixteen-week-old apo E KO mice were randomly distributed in two experimental groups: Trained 60 min (T; *n* = 29) and Sedentary (S; *n* = 32). Before starting training, animals from both groups were acclimated to treadmill exercise, but only those selected to the trained group were submitted to exercise training protocol. During acclimation, mice were placed in a treadmill for 10 min at 12 m/min with a progressive increment up to 15 m/min. The percent of mice excluded from the study because of incapacity to run accounted to 10.2%.

The T group was submitted to a 6-week aerobic exercise training (AET) protocol on a treadmill, 5 days/week. In the first week, the mice were exercised on the treadmill at a speed of 12 m/min with a gradual increase in time session from 30 to 60 min. Thereafter, running was performed at 15 m/min during 60 min. This protocol was chosen for its efficiency in reducing the area of advanced atherosclerotic lesion and increase markers of plaque stability according to Kadoglou et al. (2013).

### Treadmill exercise test

Treadmill exercise test was performed after mice acclimation to exercise. Treadmill speed started at 6 m/min at 0% grade, and every 3 min was adding 3 m/min until exhaustion (Ferreira et al., 2007). Exhaustion was determined by the inability to maintain the running pattern. The exercise capacity was analyzed by time to exhaustion and Δ time to exhaustion (time to exhaustion basal—time to exhaustion after physical training) in test.

### Sample collection and biochemical analysis

Plasma TG and TC were measured by enzymatic colorimetric kits obtained from Labtest (MG, Brazil). HDL-c was determined by measurement of cholesterol after selective precipitation of the LDL and VLDL particle in the plasma (Labtest, MG, Brazil). Glucose was determined by Accu Check Performa glucometer. All determinations were carried out in plasma samples drawn after a 12 h overnight fasting period. Twenty-four hours after the last exercise session and just before euthanasia, blood was collected by the caudal vein (approximately 600 μL) in tubes containing heparin and butylated hydroxytoluene (BHT; 5 μg/mL). Plasma was immediately obtained by centrifugation (775 × g for 10 min) and stored at −80°C. Mouse euthanasia was carried out using a CO_2_ chamber with a gradual fill method (displacement rate from 10 to 30% of the chamber vol/min). The aortic arch was infused with ice-cold 0.9% NaCl, excised in the fresh state, and preserved in −80°C for oxysterol and mRNA evaluation.

### Lipoproteins isolation and LDL acetylation

Procedures with humans were in accordance with the Declaration of Helsinki. All blood donors had signed an informed written consent form previously approved by The Ethical Committee for Human Research Protocols of the Hospital das Clinicas HCFMUSP, Faculdade de Medicina, Universidade de São Paulo, São Paulo (CAPPesq # 773/06 and 441/11). LDL (*d* = 1.019–1.063 g/mL) and HDL_2_ (*d* = 1.125–1.21 g/mL) were isolated from healthy plasma donors by sequential ultracentrifugation and further purified by discontinuous gradient ultracentrifugation. Protein content was determined by the Lowry et al. (1951) procedure. LDL acetylation was performed according to Basu et al. (1976). After extensive dialysis against ethylenediaminetetraacetic phosphate-buffered saline, acetylated LDL and HDL were maintained sterile at 4°C under N_2_ atmosphere and used within a month.

### Cell culture and AMPK activation

J774 macrophages were cultured in RPMI 1640 medium (Gibco, Grand Island, New York, USA) containing 10% fetal calf serum (Cultilab, Campinas, Brazil), penicillin and streptomycin (Gibco), and maintained in a 5% CO_2_ incubator at 37°C. After reaching confluence, macrophages were enriched with acetylated LDL (75 μg/mL DMEM) for 48 h and after washing treated with 0.25 mM 5-aminoimidazole-4-carboxamide 1-β-D-ribofuranoside (AICAR; Sigma-Aldrich, St. Louis, USA) or DMEM alone for 24 h. At the end of the incubation, cells were washed and incubated with of HDL_2_ (50 μg/mL DMEM), for 6 h.

### Cholesterol and oxysterol measurement by gas chromatography-mass spectrometry (CG/MS)

Lipids were extracted from the aortic arch with 6 mL chloroform/methanol (2:1; v:v) and 2 mL water containing butylated hydroxytoluene (BHT; 5 μg/mL). The homogenized content was then centrifuged at 1,690 × g for 15 min, 4°C; the aqueous phase was removed and the organic phase vacuum-evaporated (Genevac EZ-2 plus, Ipswich, England). Cellular lipids were extracted with 6 mL hexane:isopropanol (3:2; v:v) and evaporated. The same procedure described below was used for the measurement of oxysterols in the aorta and macrophage lipid extracts and in plasma. A mixture of deuterium-labeled internal standard (7α-hydroxycholesterol-d7, 7β-hydroxycholesterol-d7, 7-Ketocholesterol-d7, 25-hydroxycholesterol-d7, 27-hydroxycholesterol-d7 and cholesterol-d7; 100 ng of each oxysterol and 20 μg of cholesterol, diluted in ethanol; Avanti Polar Lipids, Alabaster, USA) was added to aorta and macrophage lipid extract and to 500 μL of animal's plasma. Due to limitation of plasma volume, a pool of plasma from two animals was utilized (250 μL of each mouse).

The measurement of total cholesterol (free + esterified) and total oxysterol subtypes (free + esterified) was performed after alkaline saponification by adding a mixture of 10 mL of absolute ethanol and 0.4 M of potassium hydroxide, for 2 h. One-hundred microliter of phosphoric acid was added followed by 20 mL of chloroform and 6 mL of water. After vigorous shaking and centrifugation (750 × g for 15 min at 4°C), the aqueous phase was removed and the organic phase vacuum-evaporated. The lipid extract was dissolved in 1 mL of toluene. Oxysterols were isolated from cholesterol by solid phase extraction (Sigma-Aldrich Supelclean LC-Si SPE Tubes SUPELCO, Bellefonte, USA). Briefly, the sample was applied into the column previously conditioned with 2 mL of hexane, following washing with 1 mL of hexane. Total cholesterol was eluted in separated tubes with 8 mL of 1.5% isopropanol in hexane, and oxysterols further eluted with 6 mL of 30% isopropanol in hexane. Finally, the solvent was evaporated and samples were derivatized with 100 μL of pyridine and 100 μL of N,O-bis (trimethylsilyl) trifluoroacetamide with trimethylchlorosilane (BSTFA; Sigma-Aldrich, St. Louis, USA), for 1 h at 60°C. One microliter of the derivatized sample was injected into a gas chromatograph coupled to a mass spectrometer (Shimadzu GCMS-QP2010, Kyoto, Japan) by automatic injector and analyzed in selected ion monitoring. The separation was performed on a Restek capillary column (100% dimethyl polysiloxane—Rxi®-1 ms. Cat. #13323), 30 m, internal diameter 0.25 mm, for 30 min, using helium as mobile phase, with constant linear velocity of 44.1 cm/sec. For oxysterols analysis, the oven started at 240°C with increment of 5°C/min, for 7 min up to 290°C. For the cholesterol analysis, the oven temperature was maintained at 260°C. The mass spectrometer operated in impact electron mode at an ionization voltage of 70 eV with the temperature of the ion source at 300°C. The quantification was performed by comparing the peak areas of the standard curve and corrected by internal standards, as previously described by Dzeletovic et al. (1995). Oxysterols and cholesterol were identified with a limit of quantification of 15 ng and 5 μg in the total sample, respectively. The intra-assay coefficient of variation was 19 and 6%, respectively, for oxysterols and cholesterol, based in six repetitive measurements of sterols in a control plasma sample. The amount of oxysterols and cholesterol was corrected per microgram of cellular or aortic protein, and per microgram of total cholesterol in plasma.

### Gene expression analysis

Total RNA was isolated from aortic arch and macrophages by using Qiagen RNeasy Mini kit as described in the manufacturer's protocol. Reverse transcription was performed using the high-capacity RNA-to-cDNA kit (Applied Biosystems). Before cDNA synthesis, quantitative and qualitative RNA evaluations were carried out with Agilent RNA 6000 Nano Kit. mRNA expression was measured by real-time quantitative polymerase chain reaction (RT-qPCR) using a gene-specific fluorogenic TaqMan probes (Applied Biosystems) in Step One Plus Real Time qPCR System (Applied Biosystems). The relative quantitation of gene expression was calculated by using the comparative cycle threshold (Ct; 2^−ΔΔCt^) method (Livak and Schmitgen, 2001). β-actin was used as housekeeping gene for both aortic tissue and macrophages.

### Statistical analysis

The unpaired Student *t*-test or Mann Whitney, according to normality of the data, was used for comparison between groups. The One-way analysis of variance (ANOVA) followed by the *post-hoc* Newman-Keuls test was utilized in order to analyze treadmill exercise test and oxysterol levels in macrophages treated with AMPK inductor. All data were checked for normality by Shapiro-Wilk test prior to statistical analysis. Data are expressed as mean ± standard deviation (*SD*) and a *p*-value < 0.05 was considered statistically significant.

## Results

Body weight and plasma TC, TG, HDL-c, and glucose were similar between T and S groups, at baseline and after intervention (Table 1). Food consumption was higher in T as compared to S (Table 1).

**Table 1:** Body weight, plasma lipids and glucose and food consumption in trained (T) and sedentary (S) mice.

		**S (*n* = 32)**	**T (*n* = 29)**
Body weight (g)	Basal	27.1 ± 2.9	26.7 ± 2.2
	Final	27.8 ± 3.3	27.6 ± 2.9
TC (mg/dL)	Basal	474 ± 100	453 ± 88
	Final	431 ± 126	438 ± 110
TG (mg/dL)	Basal	93 ± 26	97 ± 31
	Final	99 ± 22	103 ± 32
HDLc (mg/dL)	Basal	20 ± 7	19 ± 5
	Final	18 ± 5	17 ± 6
Glucose (mg/dL)	Basal	107 ± 14	110 ± 16
	Final	98 ± 12	102 ± 16
Food consumption		4.1 ± 0.6	4.5 ± 0.5^*^
(g/animal/day)		(*n* = 11)	(*n* = 11)

*Data expressed as mean values ± standard deviation were compared by the Student's t test. CT, total cholesterol; HDL-c, HDL cholesterol; TG, triglycerides*.

* p < 0.05

**Figure 1:**
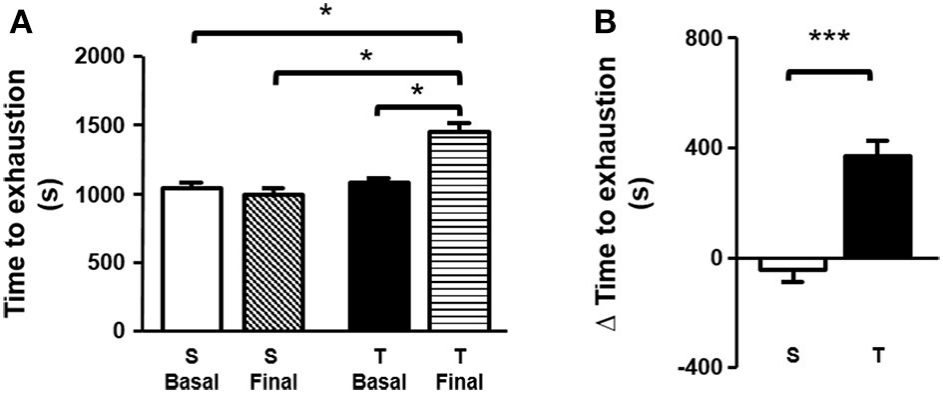
**(A)** Time to exhaustion (basal and final). Time to exhaustion was compared by one-way ANOVA with Newman-Keuls post-test. **(B)** Δ time to exhaustion in treadmill exercise test. Δ time to exhaustion was analyzed by unpaired Student's t test. Data expressed as mean ± SD. Sedentary group (S), *n* = 32; trained group (T), *n* = 29. ^*^p < 0.05; ^***^p < 0.001.

Time to exhaustion determined in treadmill exercise test (expressed as seconds or delta time), was higher in T animals as compared to S. Furthermore, the trained mice improved exercise tolerance when compared to the baseline period (Figures 1A,B). No significant changes were found in the S group.

In plasma, the most prevalent oxysterols were 7-KC and 7β-OH C, representing 34 and 29%, respectively, of the total oxysterol amount. Plasma concentrations of 7α-OH C, 27-OH C, and 25-OH C were similar, representing 18, 12, and 7%, respectively, of total plasma oxysterols. Exercise training did not change plasma oxysterol concentration and their proportions in T as compared to S group (Table 2).

**Table 2:** Oxysterol levels in plasma and aortic arch of trained (T) and sedentary (S) mice.

	**Plasma (ng/mg of cholesterol)**		**Aortic arch (ng/mg protein)**	
	**S (*n* = 8)**	**T (*n* = 8)**	**S (*n* = 5)**	**T (*n* = 4)**
7α-OH C	47.7 ± 26.1	36.4 ± 14.9	43.3 ± 16.1	61.2 ± 16.6
7β-OH C	72.5 ± 36.9	57.8 ± 12.7	31.2 ± 4.4	53.0 ± 7.7^*^
7-KC	79.3 ± 30.4	76.2 ± 27.0	116.0 ± 48.1	99.1 ± 15.4
25-OH C	14.6 ± 3.3	15.1 ± 2.1	ND	ND
27-OH C	30.6 ± 14.6	26.3 ± 9.1	ND	ND
Total	246.2 ± 43.36	211.6 ± 18.08	190.5 ± 46.7	213.3 ± 42.7

*Data expressed as mean values ± standard deviation were compared by the Student's t test. 7α-OH C, 7α-hydroxycholesterol; 7β-OH C, 7β-hydroxycholesterol; 7-KC, 7-ketocholesterol; 25-OH C, 25-hydroxycholesterol; 27-OH C, 27-hydroxycholesterol; ND, not determined*.

* *p < 0.05*.

**Figure 2:**
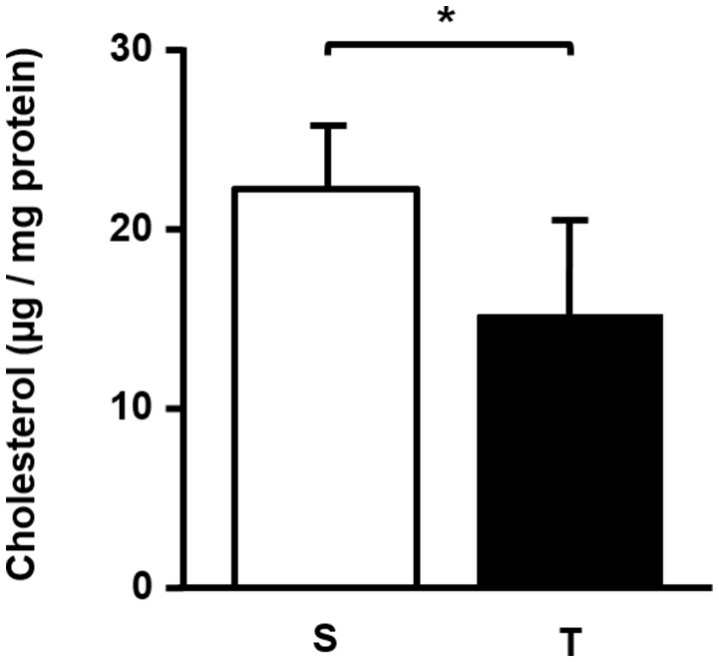
Total cholesterol (μg/mg protein) in the aortic arch of trained and sedentary mice. Data expressed as mean ± *SD* were compared by the Student's *t*-test. Sedentary group (S), *n* = 5; trained group (T), *n* = 4. ^*^p < 0.05.

In the aortic arch, there was a higher amount of 7β-OH C in T mice compared to S mice (Table 2). 7-KC and total oxysterol level in the aortic arch were similar between T and S mice. 7-KC levels corresponded to 53% of total oxysterols, followed by 7β-OH C (21%). 27-OH C, and 25-OH C were not detected in the arterial wall. There was a significant reduction in cholesterol levels after 6-weeks of AET in T group as compared to S group (32%; Figure 2).

**Figure 3:**
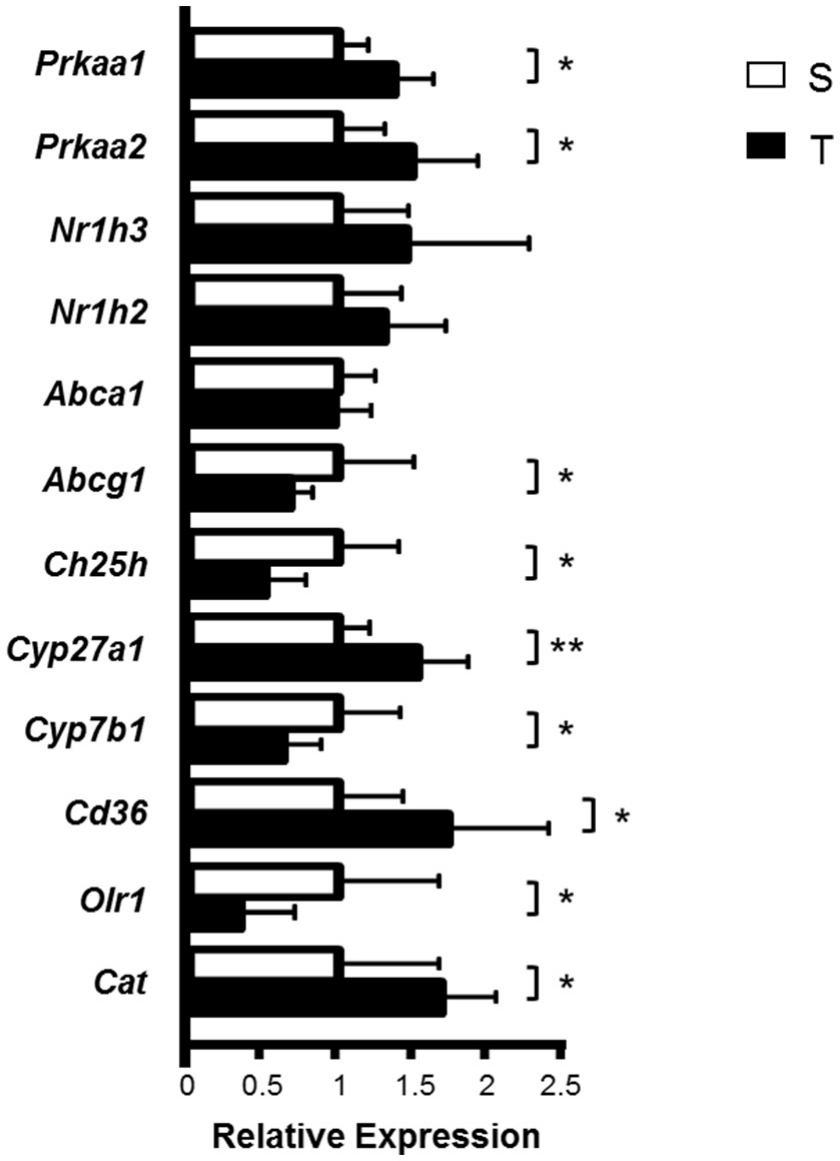
Relative expression of genes involved in cholesterol and oxysterol homeostasis in the aortic arch of trained and sedentary mice. Aortic arch was isolated from S (*n* = 8) and T (*n* = 6) mice 24 h after the last exercise session. mRNA expression was measured by RT-qPCR using a gene-specific fluorogenic TaqMan probes (Applied Biosystems): *Abca1* Mm00442646_m1; *Abcg1* Mm00437390_m1; *Cat* Mm01340247_m1; *Cd36* Mm01135198_m1; *Ch25h* Mm00515486_s1; *Cyp27a1* Mm00470430_m1; *Cyp7a1* Mm00484150_m1; *Cyp7b1* Mm00484157_m1; *Nr1h2* Mm00437265_g1; *Nr1h3* Mm00443451_m1; *Olr1* Mm00454586_m1; *Prkaa1* Mm00443451_m1; *Prkaa2* Mm01264789_m1. Relative quantification of gene expression was calculated by using the comparative cycle threshold (Ct; 2^−ΔΔCt^) method. *Actb* (Mm00607939_s1) was used as housekeeping gene. Data expressed as mean ± *SD* were compared by the Student's *t*-test. ^*^p < 0.05; ^**^p < 0.01.

The expression of *Prkaa1* (AMPK α1) and *Prkaa2* (AMPK α2)—key proteins in cellular energy metabolism activated by exercise; *Cyp27a1*—an enzyme that produces 27-OH C; *Cd36—*a scavenger receptor and *Cat* (catalase)—an antioxidant enzyme, was higher in T mice as compared to S (Figure 3). On the other hand, AET reduced the expression of *Abcg1*—a transporter of cholesterol and oxysterol to HDL; *Ch25h*—an enzyme that produces 25-OH C; *Cyp7b1*—an enzyme that metabolizes 27-OH C, and *Olr1* (LOX-1)—a scavenger receptor that mediates the uptake of oxidized LDL (Figure 3). *Abca1, Nr1h3 (LXR*α*)* and *Nr1h2 (LXR*β*)* mRNA were not modified by exercise training in the aorta (Figure 3).

**Figure 4:**
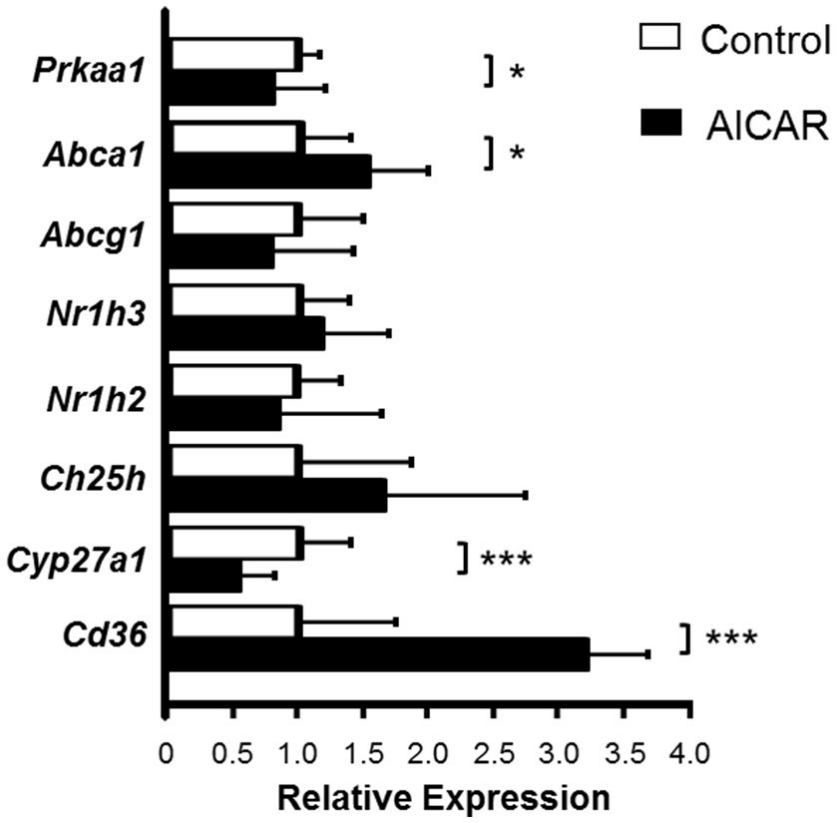
Relative gene expression of the genes involved in cholesterol and oxysterol homeostasis in macrophages treated with AMPK inductor. J774 macrophages were enriched with oxidized LDL (75 μg/mL) for 48 h and then treated in the absence or presence of 0.25 mM AICAR, for 24 h. mRNA expression was measured by RT-qPCR using a gene-specific fluorogenic TaqMan probes (Applied Biosystems): *Abca1* Mm00442646_m1; *Abcg1* Mm00437390_m1; *Cd36* Mm01135198_m1; *Ch25h* Mm00515486_s1; *Cyp27a1* Mm00470430_m1; *Cyp7a1* Mm00484150_m1; *Cyp7b1* Mm00484157_m1; *Nr1h2* Mm00437265_g1; *Nr1h3* Mm00443451_m1; *Olr1* Mm00454586_m1; *Prkaa1* Mm00443451_m1; *Prkaa2* Mm01264789_m1. *Cyp7a1, Cyp7b1, Olr1, Prkaa2* genes were very low or not expressed in this cell type. Relative quantification of gene expression was calculated by using the comparative cycle threshold (Ct; 2^−ΔΔCt^) method. *Actb (Mm00607939*_s1) was used as housekeeping gene. Data expressed as mean ± *SD* were compared by the Student's *t*-test. Control *n* = 7; AICAR *n* = 6. ^*^p < 0.05; ^***^p < 0.001.

Considering the increase in *Prkaa1* and *Prkaa2* mRNA by AET, the effect of AMPK activation in macrophage oxysterol levels was evaluated. The J774 macrophages were cultured in the absence or presence of 0.25 mM AICAR (AMPK activator), for 24 h and subsequently incubated in the absence or presence of HDL for 6 h, in order to stimulate sterol efflux. The AICAR concentration used represented the highest concentration that does not induce decrease in cell viability, which was observed by lactate dehydrogenase release in the medium (data not show). AMPK activation in the absence of HDL did not alter the concentration of cellular sterols compared to control cells (Table 3). However, the treatment with AICAR and HDL_2_ decreased the macrophage concentration of 7α-OH C but did not change the levels of 7β-OH C, 7-KC, and TC (Table 3). In addition, AICAR treatment increased *Abca1* and *Cd36* and reduced *Prkaa1* and *Cyp27a1* expressions. *Abcg1, Nr1h,3* and *Nr1h2* expressions were not modified by the AMPK activation (Figure 4).

**Table 3:** Oxysterol levels (ng/mg protein) and cholesterol (μg/mg protein) in macrophages treated with AMPK inductor.

	**Control (*n* = 7)**	**AICAR (*n* = 7)**	**Control + HDL_2_ (*n* = 7)**	**AICAR + HDL_2_ (*n* = 7)**
7α-OH C	4.7 ± 2.3	4.7 ± 2.1	11.4 ± 4.9	6.1 ± 1.8^*^
7β-OH C	8.4 ± 2.1	7.4 ± 4.1	14.1 ± 4.8	10.4 ± 2.6
7-KC	53.1 ± 11.3	42.4 ± 14.4	74.8 ± 27.8	53.2 ± 17.8
Cholesterol	34.6 ± 13.5	37.6 ± 13.5	46.6 ± 20.4	43.9 ± 18.8

*J774 macrophages were treated with 75 μg/mL acLDL for 48 h. After washing, the cells were maintained 24 h in supplemented medium, or not, with 0.25 mM AICAR (Sigma-Aldrich, St. Louis, USA) for activation of AMPK. Then, in some incubations, 50 μg/mL of HDL in DMEM (6 h) was used in order to stimulate sterols efflux. Oxysterol levels were measured by GC-MS. The results (mean ± SD) were compared by one-way ANOVA with Newman-Keuls post-test. 7α-OH C, 7α-hydroxycholesterol; 7β-OH C, 7β-hydroxycholesterol; 7-KC, 7-ketocholesterol*.

* p < 0.05—Control + HDL_2_ vs. AICAR + HDL_2._

## Discussion

AET has beneficial effects on lipid metabolism, including the RCT, which contributes to the prevention of atherosclerosis (Rocco et al., 2011; Kadoglou et al., 2013). Considering the role of oxysterols in modulating cell lipid homeostasis and inflammation in the arterial wall, we investigated in the apo E KO mice the role of a 6-week AET program in oxysterol and cholesterol accumulation in the aortic arch and plasma, and the expression of genes related to their metabolism in the aortic arch.

The amount of cholesterol and oxysterols is regulated in macrophages by interplay of mechanisms including: the uptake of oxidized LDL, enzymatic and non-enzymatic—mediated synthesis and conversion and cell exportation through passive diffusion and active mechanisms. In this investigation, mice fed a chow diet avoiding the influence of high fat/cholesterol diets as an external source of oxysterols that may be carried by lipoproteins.

Even in the absence of changes in plasma TC, HDL-c, and oxysterols in dyslipidemic mice, AET increased the aortic arch concentration of 7β-OH C. This sterol can be obtained by the macrophage uptake of oxidized LDL rather than intracellular cholesterol oxidation, which in accordance to the enhanced expression of *Cd36* observed in trained mice. Despite that, exercise training reduced TC content in the aortic arch—that dictates plaque development and correlates with lesion area—and increased *Cat* mRNA. These findings agree to the role of AET in increasing RCT and reducing atherosclerotic lesion development as previously reported (Meilhac et al., 2001; Rocco et al., 2011). Acute exercise is associated with an increase in oxysterols. In contrast, chronic exercise has been shown to increase of antioxidant defenses such as, catalase and nitric oxidase synthase (Meilhac et al., 2001; Cardenia et al., 2017). Thus, catalase seems to counteract the increase in oxysterols obtained by oxLDL and cholesterol oxidation, which favors the reduction in atherosclerotic lesion.

Enhanced transcriptional rate of *Cd36* after exercise training have been described in humans and animal models as a mechanism for lipid clearance associated with RCT (Butcher et al., 2008; Pinto et al., 2015; Ruffino et al., 2016). In the present investigation, AET distinctly influenced the expression of *Cd36* and *Olr-1* (LOX1), respectively, increased (75%) and diminished (65%).

To our knowledge, few studies evaluated the influence of exercise training in the formation of oxysterols, with divergent results. Cardenia et al. (2017) observed in rat that acute exercise until exhaustion increased hepatic concentrations of 7α-OH C, 7β-OH C, 7-KC, and 27-OH C, that was prevented by 45 days of rich broccoli extract enriched-diet. On the other hand, Musman et al. (2016) showed that physical training for 4 weeks reduced oxysterols in plasma and isolated mitochondria of ob/ob mice undergoing ischemia/reperfusion. This was due to the decrease of cholesterol content in the mitochondria and to the increase of the antioxidant capacity of the cardiac tissue. Exercise protocol (acute vs. chronic), exercise intensity, and tissue analysis may explain the difference across studies.

Enhanced and decreased expressions of, respectively, *Cyp27a1* and *Cyp7b1* mRNA, may contribute to the enhancement of 27-OH C, although no detectable levels of 27-OH C were observed in our CG/MS analyses. Low or absent of 27-OH C was also reported in apo E KO mice aorta and macrophages by, respectively, Vaya et al. (2001) and Maor et al. (2000). This may be attributed to the rapid flow of 27-OH C outside cells as an alternative pathway for the RCT, as previously described (Björkhem et al., 1994). Björkhem et al. (1994) observed that macrophages eliminate large amounts of cholesterol as 27-OH C by passive diffusion and Lange et al. (1995) demonstrated that the transfer of 25-OH C from the red blood cell to plasma acceptors occurs 2,000 times faster than cholesterol. Similarly, the spontaneous exchange rate between phospholipids vesicles was more efficient for 7-oxysterols, such as, 7β-OH C, compared to cholesterol (Kan et al., 1992).

In humans with hypoalphalipoproteinemia increase in plasma concentration of 27-OH C has been reported, which may overlap their reduced cell cholesterol efflux rate (Nunes et al., 2014). Independently of genetics and metabolic fate, the HDL cholesterol range in plasma is linked to a 40% variation in 27-OH C associated to HDL particle (Karuna et al., 2011). The ABCA-1 or ABCG-1 knockout bone marrow transplantation to wild type animals reduced the 27-OH C/cholesterol ratio in plasma (Karuna et al., 2011). Nonetheless, the exact role of these transporters in the efflux of 27-OH C to HDL remains unknown. In the present investigation, a reduction in *Abcg1* mRNA levels in exercised mice was found, which was interpreted as related to the reduction in cholesterol arterial levels.

By activating AMPK, AICAR systemically modulates the energetic balance and reflects the major effects of physical exercise in many organs including the liver, skeletal muscle, heart, adipose tissue, and the arterial wall compartment as well (Ruderman et al., 2003; Kim et al., 2014). Macrophages are the major cells involved in the pathophysiology of atherosclerosis. They contain scavenger receptors that uptake modified LDL leading to excess cholesterol supplying and to the inflammatory process that accompanies the development of atherosclerosis. In addition, by the RCT macrophages dictate the final amount of cholesterol in the arterial intima-media layer. Considering these aspects and the increased levels of *Prkaa1* and *Prkaa2* after exercise training in mice, we utilized J774 macrophages in a well-controlled condition in order to test the specific effects of AMPK activation on cellular sterol levels.

Similarly to the aortic arch isolated from trained mice, macrophages treated with AMPK activator presented enhanced levels of *Cd36* after incubating with HDL_2_, as an extracellular lipid acceptor. On the contrary, a reduction in *Cyp27a1* and 7α-OH C was observed, with no changes in other sterols including TC. This was related to an increase in *Abca1* mRNA expression that is reported by few authors as a transporter for oxysterols as well as free cholesterol in fibroblasts and neurons (Tam et al., 2006; Matsuda et al., 2013).

Then, the *in vitro* data do not exactly reflect the biology of the arterial oxysterols under influence of AET and AMPK cannot be defined as a mediator of the exercise effects on oxysterol metabolism and RCT in the experimental conditions utilized by us. An enhanced expression of *Abcg1*, its protein level and cholesterol efflux elicited by the AMPK activation, was previously demonstrated by others (Li et al., 2010a) but dealing with AICAR concentrations that were found by others (Santidrián et al., 2010; Russe et al., 2014; Chang et al., 2016) and us as cytotoxic. Thus, the exact role of AMPK in mediating the effects of exercise training in the arterial wall lipid homeostasis should be further investigated. Our study was limited by the lack of detectable levels of *Cyp7a1* gene expression in the aorta of the mice and *Cyp7a1, Cyp7b1, Olr1*, and *Prkaa2* in cultured macrophages. In addition, the validation of gene expression by measuring protein levels was not performed due to the limitation of the aortic arch extent.

In conclusion, in dyslipidemic mice AET increases the non-enzymatic-driven oxysterol, 7β-hydroxycholesterol, formed by cholesterol autoxidation, which is related to the enhanced expression of *Cd36*. The rapid diffusion of oxysterols, as a complementary pathway for the RCT, may also be favored by the increase and reduction of *Cyp27a1* and *Cyp7b1* expressions, respectively, which in turns favors 27-OH C desorption from cells. Together with its direct role in improving RCT as previously reported (Rocco et al., 2011), AET diminishes cholesterol accumulation in the arterial wall preventing atherosclerosis.

## Author contributions

GF performed mice exercise training, cell treatment, carried out all analysis with help of the other authors, and participated in the manuscript preparation and experimental design. PP participated in experimental design and helped in mice exercise training and RT-qPCR analysis. RI helped in cell treatment and analysis. VB helped in animal care and analysis. MS helped in oxysterols analysis. VN helped in chromatographic analysis and its data interpretations. CN had a substantial contribution to the initial concept of this paper. SC performed animal surgery, and participated in experimental design; MP was responsible for experimental design, coordination of research and preparation of the manuscript. EN helped in statistics. All authors read and approved the final manuscript.

### Conflict of interest statement

The authors declare that the research was conducted in the absence of any commercial or financial relationships that could be construed as a potential conflict of interest.
